# Low-frequency tremor-like episodes before the 2023 *M*_W_ 7.8 Türkiye earthquake linked to cement quarrying

**DOI:** 10.1038/s41598-025-88381-x

**Published:** 2025-02-21

**Authors:** Zahra Zali, Patricia Martínez-Garzón, Grzegorz Kwiatek, Sebastián Núñez-Jara, Gregory C. Beroza, Fabrice Cotton, Marco Bohnhoff

**Affiliations:** 1 Helmholtz Centre Potsdam, GFZ German Research Centre for Geosciences, Potsdam, Germany; 2 Department of Geophysics, Stanford University, Stanford, CA USA; 3 Institute of Geosciences, University of Potsdam, Potsdam, Germany; 4 Institute of Geological Sciences, Free University Berlin, Berlin, Germany

**Keywords:** Low-frequency signals, Seismicity, Kahramanmaraş Earthquake, Narlı Fault, Machine learning, Deep clustering, Geophysics, Seismology

## Abstract

Recent advances in artificial intelligence have enhanced the detection and identification of transient low-amplitude signals across the entire frequency spectrum, shedding light on deformation processes preceding natural hazards. This study investigates low-frequency, low-amplitude signals preceding the 2023 M_W_ 7.8 Kahramanmaraş earthquake in Türkiye. Using a deep neural network, we extract key features from the spectrograms of continuous seismic signals and employ unsupervised clustering to reveal distinct transient patterns. We identify an increased occurrence of low-frequency tremor-like signals during the six months preceding the mainshock. However, the location of these signals suggests that their origin is not tectonic, but rather related to anthropogenic activities at cement plants along the Narlı Fault, where the M_W_ 7.8 mainshock nucleated. Such findings highlight the importance of understanding the origin of patterns detected by machine-learning methods and the large variety of seismic signals due to anthropogenic activities. Furthermore, the search for the origin of the tremor-like signals motivated an investigation into the local seismicity around the Narlı Fault. The resulting extended seismicity catalog suggests that seismicity in this area arises from a combination of tectonic and anthropogenic processes.

**Supplementary Information:**

The online version contains supplementary material available at 10.1038/s41598-025-88381-x.

## Introduction

Recent advancements in seismic monitoring through the application of artificial intelligence and template matching to continuous waveform recordings have greatly enhanced the detection and identification of low-amplitude seismic signals. The application of machine learning (ML) to phase picking and association tasks has been particularly successful and enabled the detection of smaller seismic events, hence decreasing the magnitude of completeness^1–4^. ML has also been successful in detecting and identifying transient low-frequency patterns related to different volcanic and tectonic processes. The physical processes that these richer catalogs of signals can help to discover and understand are only starting to be explored.

Subtle deformation-related signals revealed by ML and covering the entire frequency spectrum can help to illuminate deformation processes preceding natural hazards like earthquakes (see e.g^5^. for a review^6^), and volcanic eruptions^7,8^. In the years to months before large earthquake ruptures in some transform plate boundaries, a transition from more distributed deformation towards localization around the future rupture area has been observed and proposed^9,10^. The localization of small-scale seismicity preceding larger earthquakes has been observed both in laboratory experiments (e.g^11–13^. and in earthquakes in nature (e.g^9,10,14,15^).

While holding a strong potential to advance our knowledge of earthquake physics, applying ML techniques to specific problems also requires careful attention to avoid misinterpretations that are only starting to be acknowledged. For instance, some ML approaches rely on the extraction of features to which a direct physical meaning cannot be attributed, such as when an autoencoder extracts a small number of features from high-dimensional seismic signals, enabling signal reconstruction and clustering^7^. In addition, the higher sensitivity of these methods allows the detection of many other processes other than tectonics, potentially including various anthropogenic activities^16^.

In some cases, tectonic and anthropogenic processes may not be completely disconnected. Stress perturbations from human activities related to applied subsurface activities such as gas/oil extraction (e.g^17,18^). , fluid injection into the subsurface (e.g^19–21^). or underground mining (e.g^22^). might be sufficient to reactivate faults sufficiently close to failure. Also, surface mining and quarrying activities such as those near cement plants have been proposed to alter stress loads and weaken the strength of nearby faults, particularly near the surface, thereby accelerating failure^23^. For example^24^, reported repeating low-frequency seismicity in the shallow crust in Mexico that is partially modulated by local mining activity. In addition, cement-plant-related activities have been observed to also produce narrow-banded low-frequency content in the seismic waveform recordings^25^.

On February 6th, 2023, southeastern Türkiye was struck by two devastating large earthquakes with *M*_W_ 7.8 and 7.5 (e.g^6,15^). The first mainshock nucleated approximately 20 km away from the main strand of the East Anatolian Fault Zone (EAFZ) along a northeast–southwest-oriented splay branch known as the Narlı Fault^26–32^, , before propagating towards and onto the EAFZ. Nine hours later, the second earthquake struck about 90 km northwest of the first mainshock, likely triggered by stress redistribution induced by the initial earthquake^33^. Before the 2023 *M*_W_ 7.8 Kahramanmaraş earthquake, a period of ~ 8 months of elevated seismicity around the epicenter suggested an extended preparation^6,15^. The previous largest earthquake along the East Anatolian Fault (EAF) was the 2020 Mw 6.8 Elazığ earthquake^34,35^, which was preceded by accelerated seismic activity starting one month earlier with a Mw 5.2 event on the same fault^36^.

In this study, we investigate the potential occurrence of low-frequency deformation-related signals signifying potential preparatory processes using ML on the seismic data during the years and months preceding the *M*_W_ 7.8 Kahramanmaraş earthquake and around its epicenter. By applying an unsupervised ML-based clustering, we found low-frequency tremor-like signals that are omnipresent in some time periods including six months prior to the *M*_W_ 7.8 Kahramanmaraş earthquake. A deeper analysis reveals a spatial coherency between the source of these signals and the location of quarries and cement plants located in the vicinity along the Narlı Fault. Additionally, we investigate the tectonic and anthropogenic seismicity around the Narlı Fault to understand the potential human-induced contributions to local seismic activity. Our analysishighlights the importance of understanding patterns discovered using ML-based methods and identifying signals associated with anthropogenic activities. The signals emitted by cement plants, the detection of quarry-related blasts, and the geographical proximity to the Narlı Fault open the question of whether anthropogenic activities (e.g. mass removal and quarrying) couldhave influenced stress loading along the fault. However, it is beyond this study to analyze and establish any relation between the two.

## Results

### Clustering reveals tremor-like episodes that reactivate 6 months pre-mainshock

The *M*_W_ 7.8 Kahramanmaraş earthquake was preceded by an about eight-month-long seismicity transient featuring elevated seismic activity organized in spatio-temporal clusters that display low Gutenberg-Richter *b*-values, suggesting a potential extended preparatory phase^6,15^. Previous studies have primarily focused on analyzing seismicity dominated by a broader frequency range of the seismic signal (e.g. 1–40 Hz). Our approach focuses on a narrower frequency band, filtering out earthquake events and investigating the remaining continuous signals. While this frequency band overlaps with the lower end of the previously studied range, it does not encompass the full range of high-frequency seismicity typically analyzed in earthquake studies.

We begin by computing the Short Time Fourier Transform (STFT) on daily segments of continuous seismic data, filtering it between 1 and 5 Hz. We then employ a denoising technique on the spectrogram, utilizing median filtering with specific settings as detailed in previous studies^37,38^. By “denoised”, we refer to the process of suppressing both earthquake/broadband transient signals and daily noise from the data. An example of the original and denoised spectrogram is presented in Fig. S1. Given the widespread occurrence of random earthquakes and broadband transient signals, as well as the high daily noise (as illustrated in Fig. S1, where elevated noise levels are observed primarily between 5 am and 3 pm) in many stations, unsupervised clustering without removing these signals would fail to reveal any distinct temporal low-frequency patterns or changes. The spectrograms’ key characteristics are automatically extracted by the layers within an autoencoder. An autoencoder is a specific type of artificial neural network, initially compressing and transforming data into a low-dimensional representation termed the latent or feature space. Subsequently, these feature representations are utilized to reconstruct the original data. We employ a Deep Embedded Clustering (DEC) technique that involves using the spectrograms’ latent representation for clustering purposes. Clustering is the process of grouping similar data points, and in this context, the features extracted from the autoencoder represent the compressed version of spectrograms. This methodology ensures the extraction of the most pertinent data features for the clustering task through the simultaneous optimization of feature extraction and clustering.

**Fig. 1:**
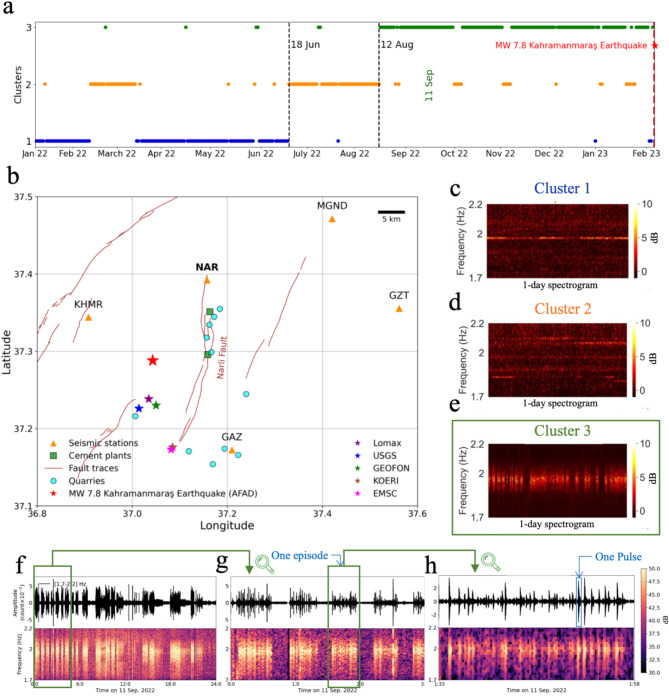
Overview of the study area and clustering results. (**A**) Deep clustering results for data from the seismic station NAR during 13 months before the 2023 *M*_W_ 7.8 Kahramanmaraş earthquake. The figure shows the results of clustering the continuous daily spectrogram of the seismic station NAR, horizontal component N, which exhibits specific patterns for different time periods. (**B**) Map of the study area showing faults and seismic stations as well as the location of local cement plants and quarries. The red star shows the epicenter of the earthquake according to the Disaster and Emergency Management Authority of Türkiye (AFAD), while the other stars indicate the epicenter as reported by different sources. Lomax epicenter location is from^39^. (**C**–**E**) A sample of spectrograms in each cluster as inputs of the clustering model. (**F**-**H**) Seismic signals and spectrograms of one day, three hours, and zoom view to one episode related to data in cluster three.

We first apply the clustering method on 14 stations located within a radius of 100 km from the future mainshock epicenter for the year 2022. Among all stations, the results show specific temporally distinct clusters (Fig. 1A) in only 5 of the closest stations within a 46 km radius around the future mainshock epicenter, all located on the eastern side of the EAFZ (Fig. 1B). The clustering analysis reveals three main clusters and two transitions around mid-June 2022 and mid-August 2022 (Fig. 1A). The clustering results are consistent across these 5 stations, indicating a common temporal pattern in the seismic data. Upon examining the data in clusters 1 and 2 (Fig. 1C & D), we observe the disappearance of a consistent, very narrow-band monochromatic signal around 2 Hz on June 18th, 2022. The spectrogram shows no specific pattern until August 12, when cluster 3 begins. Cluster 3 shows sporadic spectral changes with transient temporal episodes of tremor-like signals (Fig. 1E-H) lasting mostly between 3 and 22 min (Fig. S2) and containing enhanced radiated energy in the frequency band 1–4 Hz, with a fundamental mode around 2 Hz (in the following we refer to these as “episodes”).

Analyzing a broader time period extending from 2017 up to the *M*_W_ 7.8 Kahramanmaraş earthquake on Feb 6th, 2023 we note that these episodes also show an enhanced occurrence during the year 2018 on the station NAR (Fig. S3). However, the most prominent surge is still observed during the last 6 months (*N* = 3741 episodes picked in the station NAR) before the Kahramanmaraş earthquake, thus partially coinciding with the heightened seismic activity and *b*-value fluctuations^6,15,40^. Hence, in the next sections, we analyze their spatio-temporal properties and their relationship with seismicity in the region.

These episodes display the largest amplitudes on the seismic station NAR, which is the closest to the *M*_W_ 7.8 mainshock epicenter (15 km away) directly to the north of the Narlı Fault, but they are visible in up to five seismic stations (see Fig. S1). The spectral composition of the episodes mainly presents a narrow band signal with a fundamental mode around 2 Hz (Fig. 1F-H) and an overtone around 4 Hz (Fig. S4). The fundamental mode contains higher energy, and the overtone is only observable at the NAR station. In all stations, the episodes exhibit higher energy on the horizontal components (Fig. S5). A closer look at the waveforms from these low-frequency episodes (Fig. 1G) shows that each of them comprises numerous shorter transient pulses (Fig. 1H). These pulses could be sometimes traced and the time offset could be picked over up to five different stations. The propagation of low-frequency pulses shows a slower moveout than typical seismic events in the crust, exhibiting travel time increases of up to approximately 5 s over the 36 km maximum distance between stations (Fig. S6). We estimate the velocity of these pulses to be approximately 2 km/s, which is closer to the typical velocity of surface waves^37,41^.

## Low-frequency episodes and their temporal and energy characteristics

**Fig. 2:**
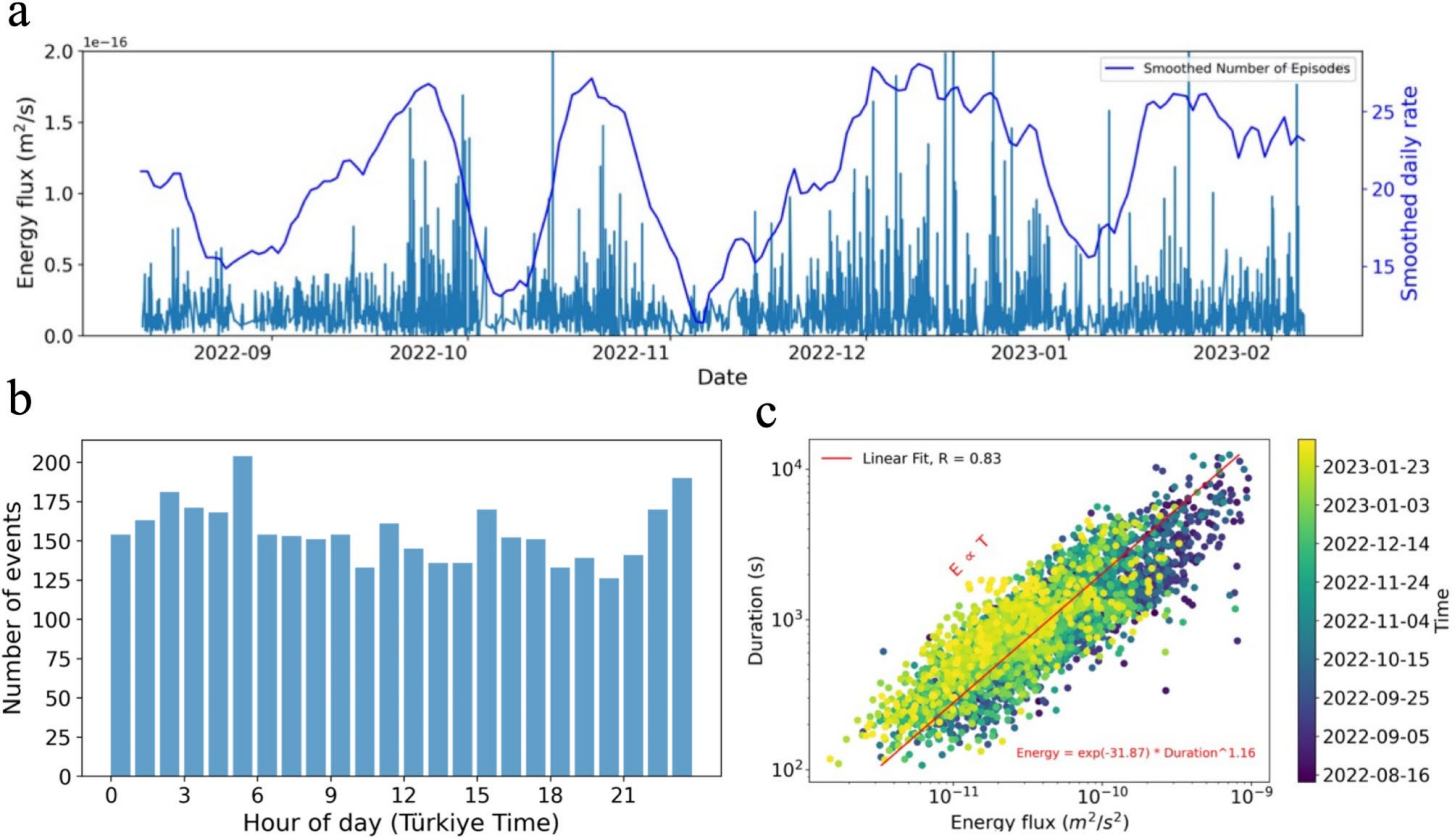
Statistical properties of the detected low-frequency episodes during the last 6 months before the 2023 *M*_W_ 7.8 Kahramanmaraş earthquake. (**A**) Evolution of energy flux and the daily number of episodes showing a relationship between them. (**B**) Histogram of the number of episodes in the hours of the day, which doesn’t show any specific hourly pattern. (**C**) The scaling law for the episodes shows an approximately linear relationship between their energy flux and duration.

In the next step, we conduct a detailed investigation on the duration, daily patterns, and energy release of episodes between 12 August 2022 and 6 February 2023. We employ a detection technique based on an amplitude threshold of a characteristic function derived from the integration of amplitudes of frequencies in each time frame of denoised spectrograms on the station NAR (see method section). Subsequently, we manually refine the start and end times of the detected episodes and add any missing events. This approach results in an accurate catalog of episodes composed of their start and end times^42^. From it, we observe that the low-frequency episodes occurred irrespective of the time of day (Fig. 2B). Unlike the low-frequency events produced by cement factories in Italy^25^, mining activity-related signals in Mexico^24^, and some of the mining-related activity events in^22^, our low-frequency episodes did not exhibit temporal distributions matching working hours on weekdays or any other specific time patterns. However, based on local information regarding cement plant activity around the Gaziantep region, these factories operate on demand up to 24 h a day. On the longer temporal scale, the occurrence of episodes from 2017 to February 2023 shows no trends such as weekly, monthly, or seasonal distribution, respectively (Fig. S3).

Furthermore, we examine the energy characteristics of these low-frequency episodes. The scaling laws governing the relationship between seismic moment and event source duration, have been explored in previous studies across a spectrum of different types of slip events^43–46^. Varied scaling laws observed for different events reflect different underlying physical processes. In regular earthquakes, the seismic moment is proportional to the cube of the duration^45^, however, this relationship may differ for slow earthquakes. Slow deformation processes observed in seismically active regions encompass a range of phenomena, including slow-slip events, tectonic tremors, episodic tremor and slip, low-frequency earthquakes, and very low-frequency earthquakes^47,48^. These events exhibit significantly longer durations compared to ordinary earthquakes with comparable seismic moments. The scaling law between seismic moment and duration for slow earthquakes has been proposed to follow a linear relationship^43–46^. Some studies have observed different relationships between moment and duration for slow events. For example^46^, identified a cubic moment-duration scaling law for slow-slip events in Cascadia, and^44^ found the same for slow-slip events in the Mexican subduction zone.

Here, to estimate the scaling law for the reported low-frequency episodes, we measure their energy flux as the squared velocity of the seismic waveform, which is corrected for instrument response, demeaned, detrended, and band-pass-filtered. As depicted in Fig. 2C, the energy flux of episodes exhibits a linear relationship with their duration, aligning more closely with those observed in slow events. We also investigate the temporal evolution in the energy flux of episodes during the last 6 months, dividing the energy flux by the duration of each episode, and comparing it to their daily occurrence rate. We observe that the variation in episodes’ energy flux shows a correlation with their occurrence rate (Fig. 2A).

### Source location and particle motion analysis of tremor-like signals point toward local cement plants

**Fig. 3:**
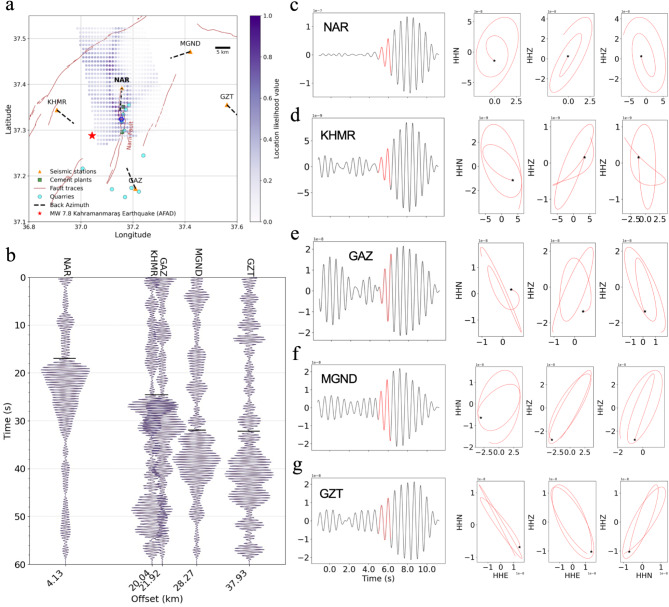
Source location analysis and particle motion of low-frequency signals (pulses) visible in various stations. (**A**) Map of the study area showing the location likelihood of pulses. The point with a circle around it marks the location with the highest likelihood. Dash lines show the resolved back azimuths of pulses in each station. The red star shows the epicenter of the *M*_W_ 7.8 earthquake according to AFAD. (**B**) Moveout of one pulse along the 5 stations filtered in [1.7–2.2] Hz, arranged based on their epicentral distance to the identified source. Small black lines mark the picked onset of an individual pulse. (**C**-**G**) Zoom on the waveforms of a particular pulse in 5 stations and their particle motion analysis projected onto the geographical planes.

In the next stage, we attempted to locate the source of the pulses. We first manually picked the first arrivals of selected 162 pulses that could be identified at 5 stations (Fig. 3B) between December 24th and December 31st, 2022. This time period was chosen due to the lower daily noise compared to other days, which allowed for more accurate picking of the first arrivals of the pulses. We found the picked offsets between arrivals on different stations to be comparable suggesting their common origin. In the following, we employ a grid-search technique^49,50^ for determining the pulse locations. We performed forward modeling of expected travel times assuming constant velocity and a predefined grid of hypocentral locations. We explore a range of velocities from 1.8 km/s (representing the lower bound of Rayleigh waves) to 3.5 km/s (representing the upper bound of S waves), and different altitudes of the hypocenter (between + 500 m and − 5000 m). For the given hypocentral location we calculated the misfit function that is dependent on the residuals between observed and calculated arrival times. An optimal model is defined that maximizes a likelihood function, and the velocity and location resulting from this model are considered the best results. Figure 3A shows the example of a likelihood map for a grid of tested hypocenters at an altitude of 500 m assuming the wave propagation velocity 1.8 km/s. This set of parameters provided an optimum stack of modeled onsets with respect to the observable ones (see Fig. S7), as well as the high values of the likelihood function trending in the south-north direction with the point with the highest location likelihood indicated by a circle in Fig. 3A. This suggests that the source of the pulse-type signals is located close to the surface, situated on or in the direct vicinity of the Narlı Fault (Fig. 3A). Closer inspection of the existing industrial infrastructure revealed two active cement plants are located along the Narlı Fault coinciding with the approximate location of the peak of the likelihood function (Fig. 3A). Furthermore, several quarries are also present along the Narlı Fault and in close proximity to both the cement plants, thus the source location of our pulse-type signals.

In the next step, we analyzed particle motions of the initial portion of manually selected pulses. The back-azimuths estimated at four seismic stations indicate the direction of incoming waves is coinciding with location of cement plants and quarries (dashed lines in Fig. 3A). However, the back azimuth in the GZT station is inconsistent with the others, likely due to the larger distance between this station and the source (38 km) and the very low signal-to-noise ratio. Both the waveform shapes (Fig. 3), as well as the particle motion analysis (Fig. 3C–G), suggest that the main type of waves composing these pulses are surface waves, possibly representing Rayleigh waves.

We found the waveforms of detected pulses are qualitatively similar to those reported in (*25*) and identified as long-period (LP) events associated with the activity of cement plants in Italy (see Fig. 2h in^25^). Our signals also resemble the waveforms and particle motion of those documented by^24^, containing most of their energy in the 1–2 Hz range and are likely related to mining activities. These comparisons support the potential link between the seismic signals we detected and the cement plant and quarrying activities along the Narlı Fault.

### A mix of quarry blasts and seismic activity around the Narlı fault

**Fig. 4:**
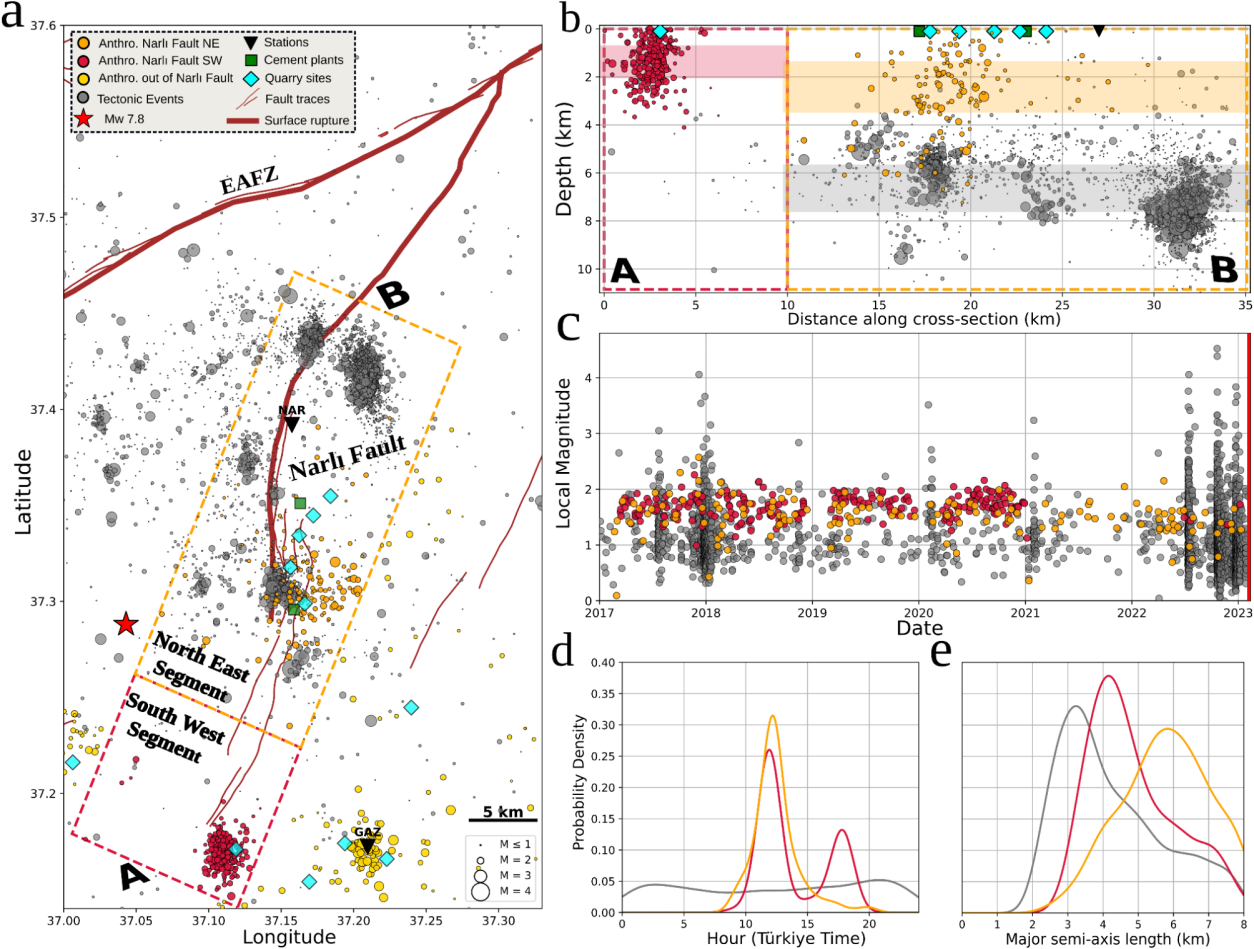
High-frequency signals around the Narlı Fault representing seismic and anthropogenic activity. Warm colors represent anthropogenic signals: red for events along the SW segment of the Narlı Fault (exclusively anthropogenic), orange for the NE segment (hosting both anthropogenic and tectonic signals), and yellow for areas farther from the Narlı Fault. Tectonic signals are shown in grey. (**A**) Map view of the anthropogenic and tectonic events around the Narlı Fault. Dashed red and orange rectangles enclose the SW and NE segments, respectively, and define the 10 km wide cross-section A–B. Bold brown lines depict the simplified M_W_ 7.8 Kahramanmaraş earthquake surface rupture, adapted from^53^, and^54^. Only events within the cross-section are analyzed in the subsequent subfigures. (**B**) Depth distribution of events along the cross-section A–B. The dashed rectangles are vertically projected into the cross-section to visualize the extent of the SW and NE segments. Vertical colored spans indicate the interquartile range of event depths for each subset. Quarry blast, cement plant, and station locations are projected onto the surface. (**C**) Magnitude-time distribution of the events. A bold red vertical line marks the onset of the M_W_ 7.8 Kahramanmaraş earthquake. (**D**,**E**) Probability density functions of (**D**) hourly distributions and (**E**) 68% uncertainty major semi-axis ellipsoid length for each subset, calculated using the non-parametric kernel density estimation.

We identified a mixture of anthropogenic and tectonic high-frequency signals on the Narlı Fault, exhibiting distinct spatiotemporal features (Fig. 4). A six-year seismic catalog (2017 to just before the Mw 7.8 earthquake on February 6th, 2023) was developed using deep learning and classical techniques. Seismic phases were picked and associated using PhaseNet^3^ and GENIE^4^, with hypocenters refined using NonLinLoc^51,52^. Only well-constrained solutions (68% semi-major axis length < 8 km and depth < 15 km) were retained. Along the Narlı Fault (rectangles in Fig. 4a and cross-section in Fig. 4b), 2866 events were manually classified, with 86% identified as tectonic (grey) and 14% as anthropogenic (red in the south, orange in the north). Classification relied on five criteria: proximity to anthropogenic sources from digital terrain maps; proximity to quarry blast clusters previously identified in regional catalogs^15^; daytime occurrence patterns shown in Fig. 4d; event depths, as anthropogenic events are typically shallower than tectonic ones, although uncertainties in hypocenter locations may affect estimates; and labeled events from the KOERI catalog.

Anthropogenic and tectonic activity segregate into distinct zones, with the exception of the area close to the cement plant activities in the NE segment of the Narlı Fault (Fig. 4a,b). In this zone (10–25 km in the cross-section of Fig. 4b), shallow anthropogenic activity is observed with significant spatial scattering due to higher hypocentral uncertainties (Fig. 4e). Slightly deeper within the same zone, clusters of tectonic events are identified. The magnitude distribution of anthropogenic events is narrower compared to tectonic events, which exhibit marked mainshock-aftershock sequences absent in anthropogenic activity (Fig. 4c).

The temporal patterns of anthropogenic activity are distinct. While both the anthropogenic events in the SW and NE Narlı Fault segments peak around noon, the SW segment also exhibits an additional peak between 17.00 and 19.00 (Fig. 4d). The SWcluster’s activity diminishes by the end of 2020 and has better-constrained hypocenters, likely due to its proximity to the GAZ station (Fig. 4e). In contrast, the NE anthropogenic cluster remains active throughout the six-year period, though its activity fades significantly by July 2022 (Fig. 4c). This decline coincides with increased tectonic activity, possibly obscuring the anthropogenic signals.

## Discussion

In this work, we refined and employed a deep clustering method^7^ to analyze the low-frequency content of continuous waveform recordings during the years before 2023 from stations close to the future epicenter of the *M*_W_ 7.8 Kahramanmaraş earthquake in Türkiye. Clustering analysis revealed tremor-like low-frequency episodes covering roughly some tens of minutes and consisting of many small pulses that became most prominent six months before the earthquake. Locating these pulses, as well as particle motion analysis indicated that the source locations of these pulses appear close to cement plants along the Narlı Fault. Our findings and their implications from three key perspectives are discussed below.

### Seismic records capture all Earth vibrations, not just tectonic

Our study highlights the importance of recognizing potential anthropogenic signals embedded in recorded seismograms. Since seismograms record any ground motions of sufficient amplitude in the Earth, regardless of their origin, it is crucial to consider that not all signals are tectonically generated and to separate waveform features from tectonic and anthropogenic origin. This awareness is vital not only for analyzing tectonic regimes and natural seismicity but also for exploring new lines of research related to anthropogenic activities. The presence of anthropogenic signals embedded in continuous seismograms and the risk of misinterpreting these as tectonic processes have been discussed in various studies. Some examples include signals related to the movement of airplanes and helicopters^55^, harmonic signals from wind turbines^56^, low-frequency tremor-like signals representing the train passage^57^, and low-frequency events produced by huge cement factories^25^. Additionally, misidentifying signals related to quarry activities with earthquakes is another common example at a higher-frequency band (e.g^57^).

Likewise, our study shows that anthropogenic signals might affect both the lower and higher parts of the frequency spectrum. On the lower frequency portion, our study identifies tremor-like episodes that are most likely associated with persistently operating machinery near cement plants. At higher frequency bands, quarry blasts may contaminate the seismicity catalogs, particularly at smaller magnitudes. These signals lead to biases in magnitude-frequency statistics and apparent clustering (cf^15^). , which can result in misinterpretations of the actual physical processes (cf^58,59^). Therefore, establishing detection criteria for surface stations might be essential to avoid confusing these signals with ones of tectonic origin. Deep learning processing techniques might be more prone than traditional seismological methods to reveal a plethora of signals with various origins (e.g. in addition to tectonics), as AI-based methods are typically more efficient in detecting signals with low amplitudes.

### AI-detected signals require thorough investigation for careful interpretation

While ML often outperforms classical techniques for many seismological tasks^60,61^, it is essential to complement these methods with further analysis to achieve a comprehensive interpretation of earth processes^1^. Studies can benefit from cross-discipline analysis; for instance, integrating GIS with seismic data can provide a better understanding of the locations and possible sources of these signals. Our study exemplifies a case where ML revealed a pattern that was not directly connected to the driving scientific hypothesis, i.e. connected to the preparatory process of the *M*_W_ 7.8 Kahramanmaraş. This underscores the importance of understanding the underlying processes highlighted by the deep learning approaches by using additional complementary data analysis. Although the patterns we discovered were not directly related to tectonic precursory signals, our further analysis allowed us to locate the signals and revealed their likely anthropogenicorigin.

Our analysis indicates that the observed tremor-like signals are unlikely to be influenced by site amplification effects based on existing studies of station response characteristics in the region^62^, Lai et al., in preparation). To support this, Fig S8 shows a Power Spectral Density (PSD) plot for the NAR station, which demonstrates that the signals fall within a consistent frequency band and do not align with known site resonance frequencies, making them unlikely artifacts of local site conditions.

### Can cement plant activities induce local seismicity?

Our study raises questions about the effect of anthropogenic industrial activity on the state of stress and local seismicity around the Narlı Fault, where at least two cement plants and many quarry sites are present (Fig. 1). Cement manufacturing involves several stages that generate significant vibrations. These stages include quarry blasts to extract raw materials, crushing of these materials, mixing different rock types, milling rocks with three conical rollers on a rotating mill, and calcination in large rotary kilns. Subsequently, cement milling takes place, where clinker (a semi-product) is ground in large mill chambers with steel balls. Some of these processes, such as certain milling operations, are conducted underground to minimize outdoor effects, which may enhance ground coupling and affect seismic signals. Unfortunately, detailed information on production strategies, including the specifics of quarry blasting, is often not publicly available.

For decades, some large-scale industrial anthropogenic activities have been identified to be capable of generating local stress transients, particularly near the surface (see^63^ for a comprehensive review). Some examples of such activities include dam impoundment, groundwater extraction, and changes in the water level (e.g^64^). , or mega infrastructure construction (e.g^65^). These activities can cause significant stress variations that, when combined with tectonic stress, may bring a fault closer to failure. In particular, the stress changes that occur in mining environments or reservoir impoundments are directly related to adding or removing a certain volume of rock or water.

Rock quarrying activities related to the process of making cement also involve mass removal from the Earth’s surface, thus they might also be able to affect the state of stress of local faults. Around the Narlı Fault, long-term systematic subsidence linked to significant mass removal from mining activity is suggested by^66^. Stress perturbation from mass removal depends on several conditions, such as the volume of the removal, the temporal scale of the activity, the distances to active faults, and the proximity of such faults to failure. However, it is important to note that the tremor-like signals detected in this study are likely caused by machinery in the cement plants rather than by quarrying activities. Therefore, these tremor-like episodes serve as direct indicators of industrial activity, but they are very unlikely to directly influence the fault stress state. The quarrying activities and their associated mass removal, are more likely to eventually be able to alter the fault stress conditions. However, estimating their potential impact remains a separate issue that requires further investigation, including a quantitative estimation of volumes of rock removed, and the modeling of different rock removal sites superimposed.

There are a few documented examples establishing a potential link between the occurrence of earthquakes and surface mass removal activities associated with quarry operations and cement plants^22,63,67,68^. One of these is the 2019 *M*_W_ 5.0 earthquake in Le Teil, France, which occurred beside a quarry site in an area without historical seismicity. By estimating the Coulomb stress change on the fault due to mass removal in the quarry^23^, concluded that these activities could have brought the fault close to a critical state and hastened the occurrence of the earthquake by more than 18,000 years. In that study, they estimated the cumulative maximum Coulomb stress change on the fault due to the total mass subtraction to be 0.19 MPa over 178 years, with a mean annual removed volume of 237,507 m³/year. The shear stress value resulting from the quarry activity there exceeds what is typically considered sufficient to trigger an earthquake (0.01 MPa). The quarry-triggering hypothesis for the Le Teil earthquake has been further supported by simulation models, which have demonstrated that realistic mass removal rates can advance the failure time of a fault^69^.

The potential human-induced contribution to local seismic activity is, however, difficult to test in the case of the Narlı Fault. The local events appear to be a mixture of natural tectonic earthquakes and signatures potentially related to anthropogenic activities. The tectonic events occur predominantly at depths greater than 4 km, where the effect of surface processes should be lower, activate portions of the fault that persist during the night time, and show visible clusters in time. (Fig. 4). Distinguishing between the tectonic and the anthropogenic events is particularly difficult in this location, as it was already noted with the standard regional seismicity catalog^15^, see Supplementary Materials).

Finally, the spatial distribution of tectonic events and cement plant-related activity correlates with the surface rupture of the forthcoming Kahramanmaraş Earthquake (Fig. 4a). Could anthropogenic industrial activities related to these cement plants have influenced the nucleation of the 2023 M_W_ 7.8 Kahramanmaraş earthquake on the Narlı Fault? Although we have not gathered sufficient information to draw conclusions about this, the earthquake’s epicenter, as reported by various agencies, is approximately 10 km away and at about 8 km depth. This distance and depth may be sufficiently large to avoid being affected by stress perturbations related to cement industrial activities. However, further analysis, including modeling the stress induced on the fault from rock mass removal in the quarries (as in^23^), will be needed to shed more light on this topic.

In summary, we employed a deep clustering method to investigate the potential occurrence of low-frequency deformation-related signals in the seismic records during the years and months before the *M*_W_ 7.8 Kahramanmaraş earthquake in Türkiye. We discovered low-frequency tremor-like signals (episodes) that are most prominent during the six months before the mainshock. Further source location and particle motion analysis revealed that the source of these signals was located near cement plants and quarries around the Narlı Fault, which hosted the epicenter of the MW 7.8 Kahramanmaraş earthquake. Hence, we suggest an anthropogenic source for these signals. The ~ 10 km distance between these cement activities and the epicenter of the earthquake is sufficiently large to assume no direct link between them.

This study highlights the importance of recognizing non-tectonic signals within seismic records that could be mixed with tectonic signals. Additionally, it underscores the necessity of integrating complementary analyses alongside machine learning techniques to accurately interpret the underlying processes of the detected patterns. Furthermore, our investigation of local seismicity around the Narlı Fault using an enhanced seismicity catalog indicates that seismicity in this area could result from a mixture of tectonic and anthropogenic processes. These findings raise the question of whether years of mass removal and quarrying activity could alter stress loading on faults and impact the local seismicity.

## Materials and methods

### Denoising seismic spectrograms and preparing the input

To identify different patterns and their temporal distribution in the continuous seismic waveform, we apply clustering to the seismic signals. Previous studies have shown that clustering seismic signals using the k-means algorithm is more effective with spectrograms compared to time series^7,70^. Therefore, we used spectrograms as input for our clustering analysis. We utilize daily seismograms with a sampling rate of 100 Hz. First, the seismograms were demeaned and detrended. Then, we calculated the short-time Fourier transform (STFT) using a window length of 16,384 samples.

High levels of daily noise at most stations would complicate the analysis. Additionally, transient earthquakes need to be removed since we aim to detect low-frequency patterns, as their presence without specific daily distribution can confuse the clustering analysis. To address this, we denoised the spectrograms from both daily noise and transient signals. We employed harmonic-percussive separation techniques for seismic signals^37,38^. A horizontal median filter was applied along the time axis of the STFT to suppress the energy of transient signals. After testing different kernel sizes, we opted for a kernel size of 1,000 samples for the median filtering. This choice removed transient signals and effectively suppressed most of the daily noise energy (see Fig. S1). The effectiveness of this technique is sensitive to the selection of kernel size and filtering parameters, which may require fine-tuning for different datasets or noise environments.

As we aimed to investigate low-frequency patterns, we initially used the frequency ranges 1–5 Hz and 5–10 Hz separately. The initial clustering analysis showed that only the frequency range of 1–5 Hz exhibited specific temporal distribution patterns. Examining sample spectrograms within each cluster revealed that the clustering results were mainly influenced by narrow-band signals around 2 Hz. Therefore, to achieve more precise clustering, we narrowed the frequency band to 1.7–2.2 Hz. This narrower frequency range also helped minimize the influence of remaining noise on the clustering results. Using this frequency range, we achieved a precise temporal clustering. Visual examination of the seismic signal shows that these patterns usually have larger amplitudes on the horizontal components, particularly on the N component (Fig. S5). Therefore, for our clustering algorithm, which relies on data from a single station and a single component, we utilized the N component.

### Dimensionality reduction and feature extraction

Within the domain of machine learning, clustering typically refers to a subset of unsupervised learning techniques designed to partition data into clusters of similar objects without the requirement for known examples or labels. However, the efficacy of traditional clustering methods diminishes as the dimensionality of input data increases^71^. To address this challenge, dimensionality reduction techniques and feature engineering are employed to transform input data into a lower-dimensional feature space before clustering is performed. The selection of this feature space significantly influences clustering performance. Deep neural networks can autonomously learn features conducive to clustering, thereby enhancing the clustering of high-dimensional data^72,73^. Our approach uses a deep neural network (DNN) to reduce the dimensionality of input data. Our approach employs an unsupervised deep learning method called deep embedded clustering (DEC), utilizing the latent data representation obtained from an autoencoder for the clustering task^70,72^. Autoencoders are neural networks designed to compress input data in the encoder segment and decompress it in the decoder segment^74^. The encoder’s role is to learn a nonlinear mapping function that transforms the input data into a hidden representation. The decoder’s objective is to reconstruct the input using this hidden representation while minimizing a reconstruction loss^75^.

### Autoencoder architecture

Our network architecture comprises an autoencoder and a clustering layer (Fig. S9). The clustering layer is connected to the autoencoder’s bottleneck. We implemented a model based on our previous study^7^. However, the introduction of skip connection layers to the decoder substantially enhanced its reconstruction capability. Skip connections play a crucial role in addressing the issue of reconstructing boundary information within the feature maps^76^. They transfer indices from convolutional layers into the transposed convolutional layers allowing a more effective reconstruction. We believe that this enhanced network effectively performs dimensionality reduction and feature extraction across a wide range of datasets. We name this model AutoencoderZ^77^, highlighting its improved version compared to our previous model. It is designed to work effectively with various data types and input sizes, demonstrating its versatility and robustness. The encoder consists of four two-dimensional convolutional layers, followed by a fully connected neural network. This part maps the flattened features, extracted by the convolutional neural network layers, to a low-dimensional latent space. The decoder consists of a fully connected neural network, followed by four transposed convolutional layers, each connected by a skip connection to the corresponding convolutional layer in the encoder. For both the convolutional and fully connected layers, we employ the Exponential Linear Unit (ELU) activation function. In the decoder’s final layer, we use a linear activation function. The loss function employed for the autoencoder ($\:{L}_{R}$) is the mean squared error (MSE), calculated as the difference between the input X and the reconstructed output X$\:{\prime\:}$ for N samples:

1 $$\:{L}_{R}=\:\frac{1}{N}\:{(X-X{\prime\:})}^{2}$$

The number of daily spectrograms utilized for training and validation are 302 and 76 respectively, comprising 80% and 20% of all inputs. During model training, the trainable parameters are optimized utilizing the Adaptive Moment Estimation (Adam) algorithm^78^, which propagates the loss backward through the model after each batch iteration. The batch size determines the number of inputs processed by the model at one time. We employ an exponential decay schedule to adjust the learning rate of the optimizer, aiming to enhance model performance and reduce training time. The hyperparameters of the model remain consistent with those employed in our previous study (Table IS2 in^7^) which were set through hyperparameter tuning.

Training and validation losses exhibit an exponential decrease throughout autoencoder training, as shown in Fig. S10. The autoencoder’s proficiency in reconstructing the input from the latent spectrogram is demonstrated in Fig. S11. The details of the spectrogram pattern remain preserved after the reconstruction from the encoded salient features of the latent space. As previously noted, the addition of skip connection layers to the model significantly enhanced this capability. This indicates that during the pre-training phase, the network has successfully learned to extract a comprehensive set of features representing the elements of the signals.

### Unsupervised clustering of continuous seismic data

During the pre-training phase, the autoencoder is trained to reconstruct the output from the latent space in proximity to the input. Subsequently, the features extracted from the bottleneck of the autoencoder are utilized for the clustering task using the k-means algorithm. K-means is recognized as one of the rapid and widely used clustering methods^79^. The algorithm divides the latent space into $\:k$ clusters, where each cluster is characterized by a cluster centroid $\:{\mu\:}_{j}$. A cluster centroid, representing the center of a cluster, corresponds to the mean of all the data points within that cluster.

To determine the optimal number of clusters, we varied the number of clusters, , ranging from 2 to 9. We calculated the Calinski-Harabasz^80^ index, also known as the variance ratio criterion, which represents the ratio of the sum of between-clusters dispersion to the sum of inter-cluster dispersion (Fig. S12). Based on the elbow point observed in the Calinski-Harabasz index plot, we selected the number of clusters to be 3. This point indicates a slowdown in the decrease of the index as the number of clusters increases, suggesting 3 clusters as an appropriate choice. Subsequently, during a fine-tuning phase, the model concurrently learns feature representations and assigns clusters to the data points. Initially, the similarity between each embedded point $\:{z}_{i}$ and cluster centroids $\:{\mu\:}_{j}$ is measured using Student’s t-distribution:

2 $$\:{q}_{ij}=\frac{{(1+|{z}_{i}\left|{\left|{\mu\:}_{j}\right|}^{2}\right)}^{-1}}{\sum\:_{j}{(1+|{z}_{i}\left|{\left|{\mu\:}_{j}\right|}^{2}\right)}^{-1}}$$

The probability $\:{q}_{ij}$ represents the probability of assigning sample $\:i$ to cluster $\:j$, yielding a collection of soft class assignments. An auxiliary target distribution $\:{p}_{ij}$ is computed using the membership probability $\:{q}_{ij}$ as follows:

3 $$\:{p}_{ij}=\frac{{q}_{ij}^{2}/\sum\:_{i}{q}_{ij}}{\sum\:_{j}({q}_{ij}^{2}/\sum\:_{i}{q}_{ij})}$$

The clustering layer during fine-tuning minimizes the Kullback-Leibler (KL) divergence between the soft assignments, $\:{q}_{ij}$, and the target distribution, $\:{p}_{ij}$:

4 $$\:L=KL\left(P\:\right|\left|\:Q\right)=\sum\:_{i}\sum\:_{j}{p}_{ij}log\left(\frac{{p}_{ij}}{{q}_{ij}}\right)$$

Since $\:{q}_{ij}$ represents the membership probability of each embedded point in each cluster, it signifies the confidence level of cluster assignments. The auxiliary target distribution $\:{p}_{ij}$ helps normalize the loss contribution of each centroid, giving more weight to samples with higher confidence levels. This enables the network to learn from high-confidence cluster assignments and refine cluster centroids by minimizing the divergence between $\:{q}_{ij}$ and $\:{p}_{ij}$. Throughout iterations in fine-tuning, cluster centroids undergo refinement, autoencoder weights are updated, and the model learns more clustering-friendly features, resulting in enhanced clustering results. Following fine-tuning, the 3 clusters exhibit clear separation in the t-SNE plot. T-SNE is an unsupervised machine learning algorithm designed to visualize high-dimensional data in a two- or three-dimensional map^81^. The distinct clustering in the t-SNE space shows the effectiveness of the Deep Embedded Clustering (DEC) algorithm in extracting the most relevant features for the clustering objective.

$\:{L}_{c}=\lambda\:L$ represents the loss function of the clustering layer. λ is a hyperparameter that adjusts the weighting of the clustering layer. A λ value that is too large may distort the latent space, leading to it not accurately representing the salient features of the data. Conversely, a λ value that is too small may nullify the impact of the clustering layer. We choose a value of 0.1 for λ, as it is commonly utilized in other studies^7,70,72,82^. To optimize the clustering layer, we utilize stochastic gradient descent with a momentum of 0.9 and a learning rate of 0.01. Momentum, acting as a moving average of gradients, aids in updating the network’s weights. The weights of the autoencoder are updated every 100 iterations.

Training stops when the number of samples experiencing changes in cluster assignments drops below 0.01% of the total input data.

### Detection of tremor-like episodes

**Fig. 5:**
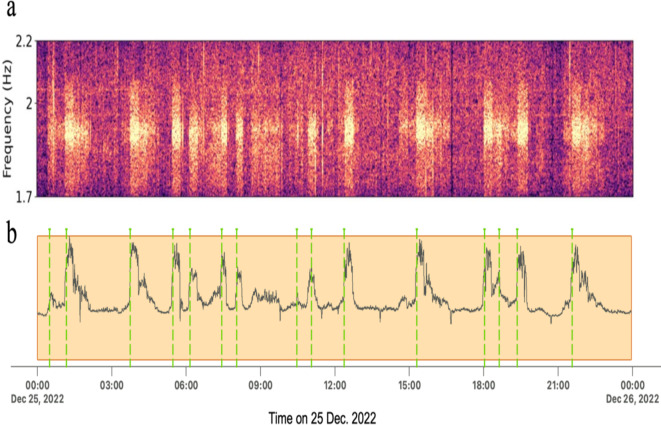
Episode detection technique. (**A**) One-day spectrogram of the HHN component at the NAR station, filtered in the [1.7–2.2] Hz band. (**B**) The characteristic function derived from the spectrogram shown in A. Dashed green lines indicate the detected peaks. The detection method is based on an amplitude threshold of a characteristic function, which is derived from integrating the amplitudes of frequencies in each time frame of denoised spectrograms^37^.

To conduct a detailed analysis of the detected tremor-like episodes, we prepared an accurate catalog of episodes, including their start and end times, from 12 August 2022 to 6 February 2023 ^42^. We used a detection technique based on an amplitude threshold of a characteristic function derived by integrating the amplitudes of frequencies in each time frame of the denoised spectrograms^37^. This characteristic function exhibits higher energy during episodes compared to other times (see Fig. 5).

To identify the start and end times of episodes, we applied a flexible heuristic to pick peaks in the normalized characteristic function^83^. In this approach, a sample $\:n$ is selected as a peak if the corresponding $\:x\left[n\right]$ that fulfills the following three conditions:

5 $$\:x\left[n\right]=max\left(x\right[n-pre\_max:n+post\_max\left]\right)$$

6 $$\:x\left[n\right]\ge\:mean\left(x\right[n-pre\_avg:n+post\_avg\left]\right)+\delta\:$$

7 $$\:n-previous\_n>wait$$

The parameters and their values, determined through our various tests, are as follows:

Pre_max: number of samples before $\:n$ over which max is computed = 8.5 min. Post_max: number of samples after $\:n$ over which max is computed = 1 min. Pre_avg: number of samples before $\:n$ over which mean is computed = 8.5 min. Post_avg: number of samples after $\:n$ over which mean is computed = 1 min. $\:\delta\:$: threshold offset for mean = 0. Wait: number of samples to wait after picking a peak = 34 min. The wait parameter helps to ensure that only one peak is picked per episode. The variable $\:previous\_n\:$represents the last sample picked as a peak. The implementation of this peak detection is based on Librosa, a Python package for audio and music signal processing^84^. The amplitude-threshold-based detection method, while straightforward, may miss episodes with low-amplitude tremor-like signals or falsely detect events due to residual noise. This method’s performance is also influenced by the choice of threshold parameters, which might vary across different stations or datasets.

## Electronic supplementary material

Below is the link to the electronic supplementary material.

Supplementary Material 1

## Data Availability

The datasets analysed during the current study are available in the Turkish National Seismic Network [https://www.fdsn.org/networks/detail/TU/]^85^ and Kandilli Observatory Network [https://www.fdsn.org/networks/detail/KO/]^86^. The code related to the proposed method is freely available in the GitHub repository [https://github.com/ZahraZali/AutoencoderZ]^77^.
