# Supplementary material for: Low-frequency tremor-like episodes before the 2023 MW 7.8 Türkiye earthquake linked to cement quarrying
Source: Sci Rep. 2025 Feb 21;15:6354. doi: 10.1038/s41598-025-88381-x (PMC11845488; doi:10.1038/s41598-025-88381-x)
Supplement: Supplementary file 1 — Supplementary Material 1 [file 41598_2025_88381_MOESM1_ESM.pdf]

Supplementary Materials for

Low-Frequency Tremor-Like Episodes Before the 2023 M_W 7.8 Türkiye Earthquake Linked to Cement Quarrying

Zahra Zali, [1]* Patricia Martínez-Garzón,[1] Grzegorz Kwiatek,[1] Sebastián Núñez Jara,[1] Gregory C Beroza[2], Fabrice Cotton[1,3], Marco Bohnhoff [1,4]

*Corresponding author. Email: zali@gfz-potsdam.de

[1] Helmholtz Centre Potsdam, GFZ German Research Centre for Geosciences, Potsdam, Germany.
[2] Stanford University, Department of Geophysics, Stanford, California, USA.
[3] Institute of Geosciences, University of Potsdam, Potsdam, Germany.
[4] Free University Berlin, Institute of Geological Sciences, Berlin, Germany.

This PDF file includes:

Figs. S1 to S12

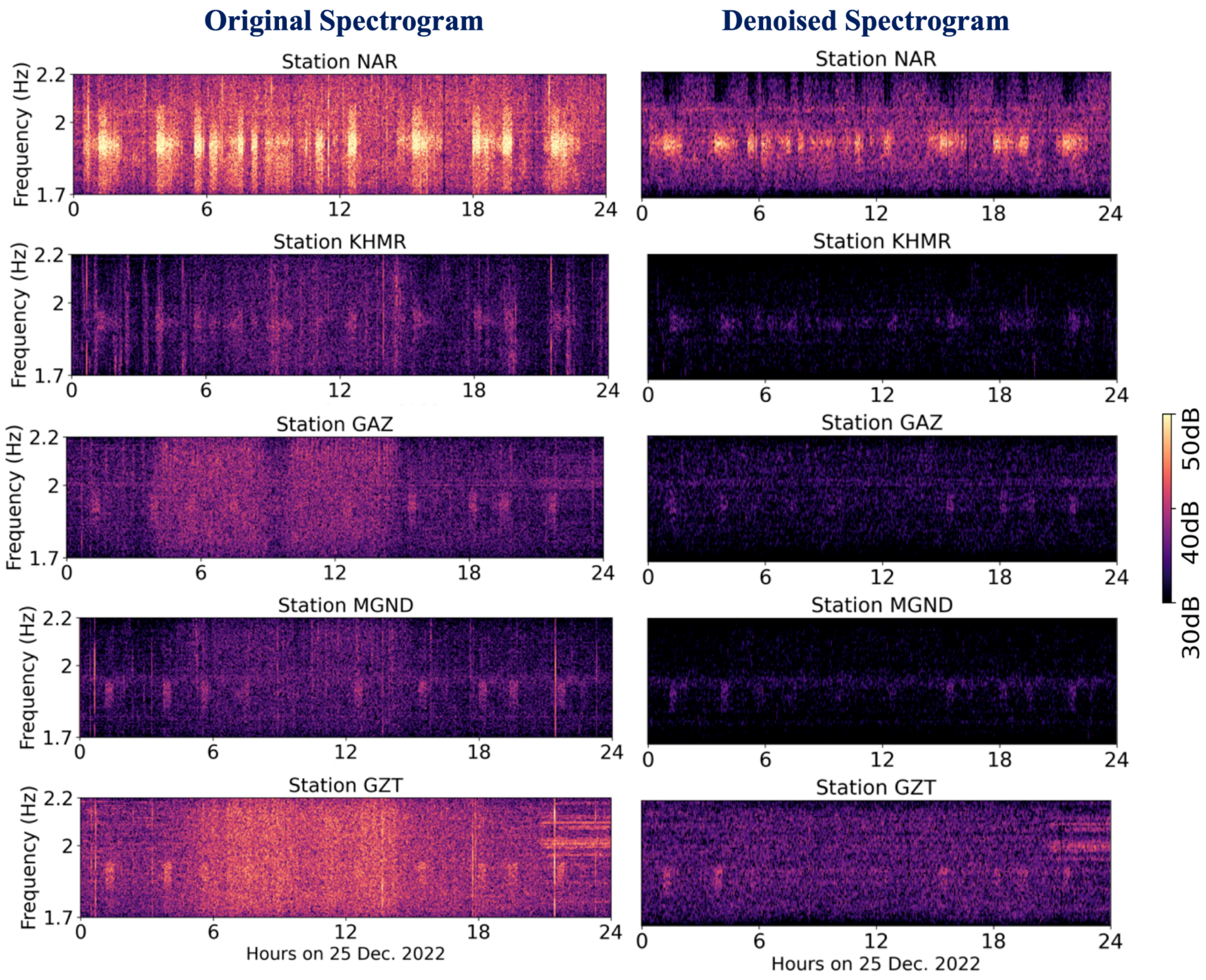

Fig. S1. Original and denoised spectrograms. Examples of the original and denoised spectrograms at the 5 stations where episodes are observed.

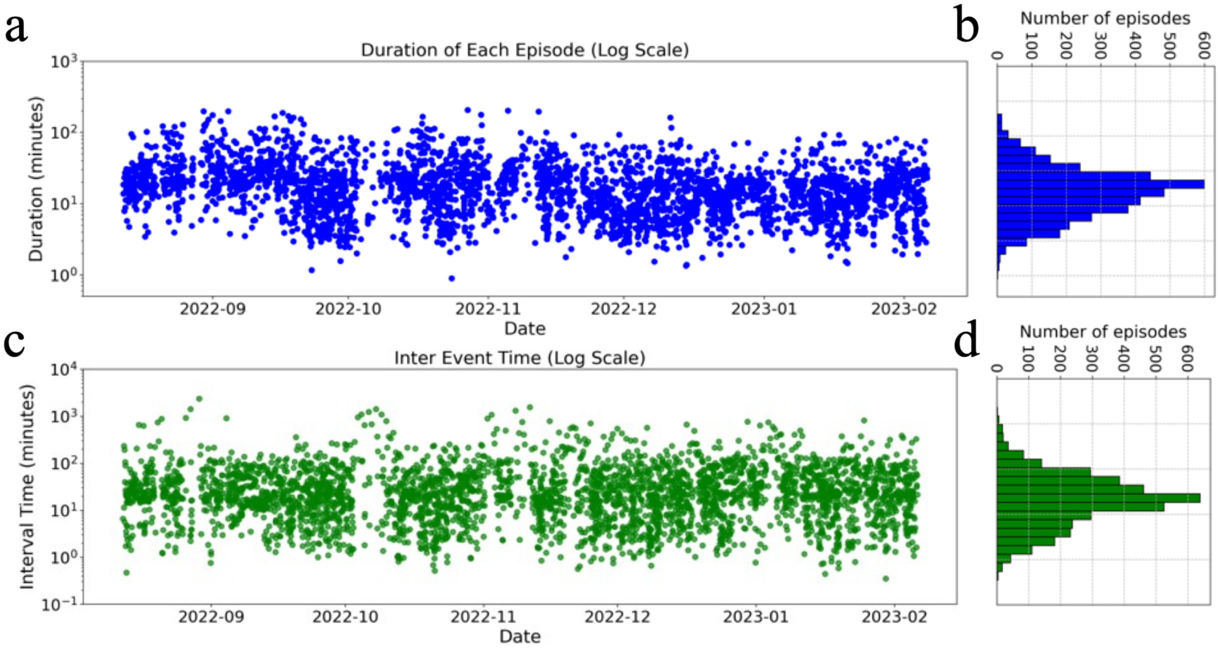

Fig. S2. Duration and inter-event time of episodes. a & **c**. The duration and inter-event time of episodes on a log scale. **c** & **d**. Histograms of duration and inter-event time of episodes, respectively.

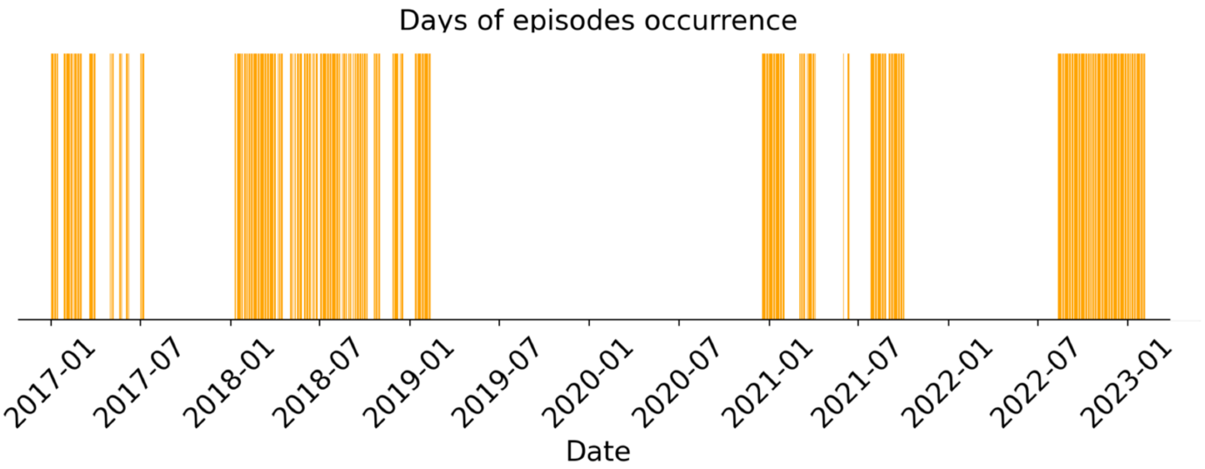

Fig. S3. Occurrence of episodes by day from 2017 to 2023.

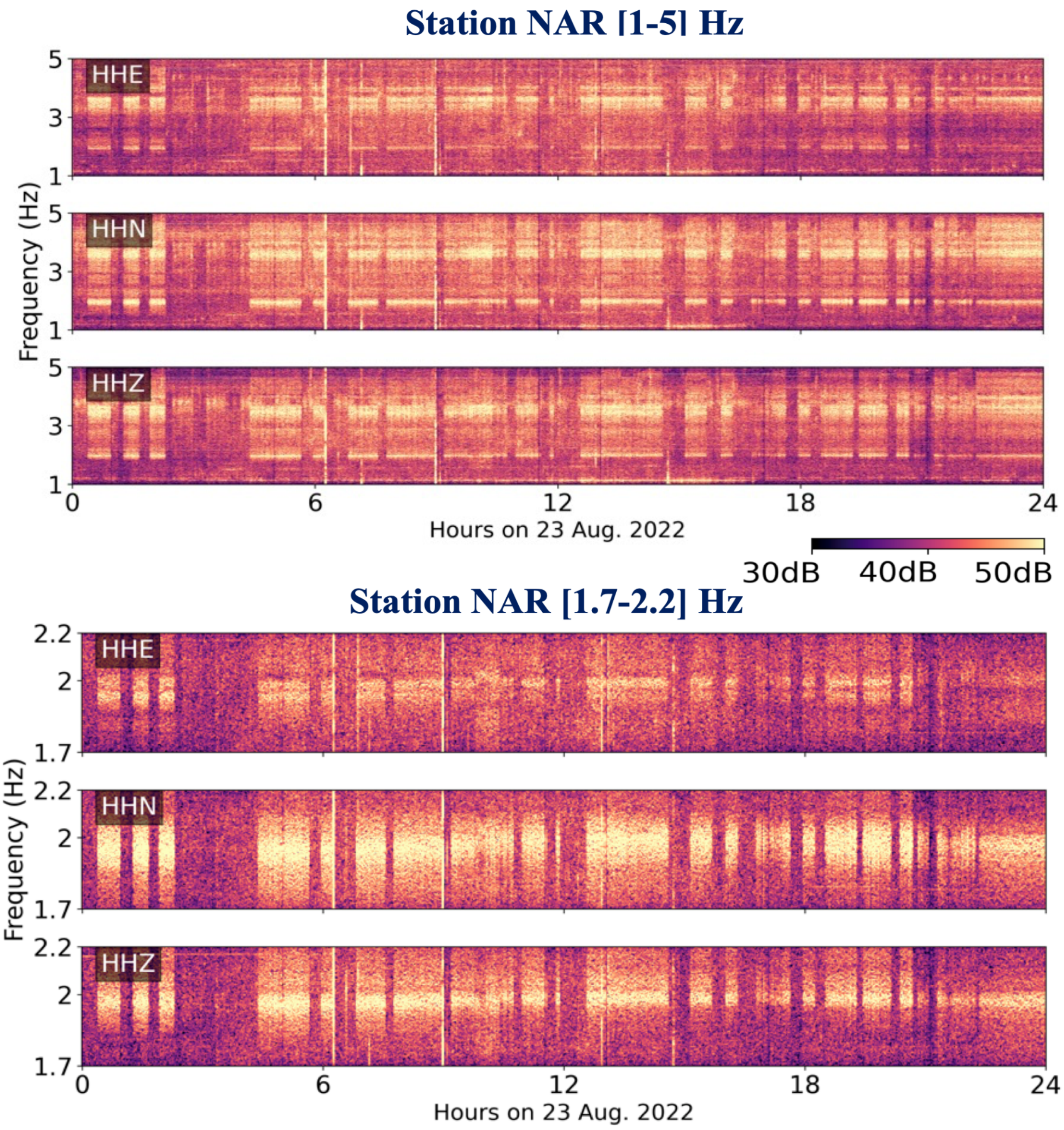

Fig. S4. Frequency content of the episodes in 2 different frequency bands. Spectrogram of one day seismic data in the frequency bands [1-5] Hz and [1.7-2.2] Hz, respectively. A fundamental mode around 2 Hz and an overtone around 4 Hz are visible.

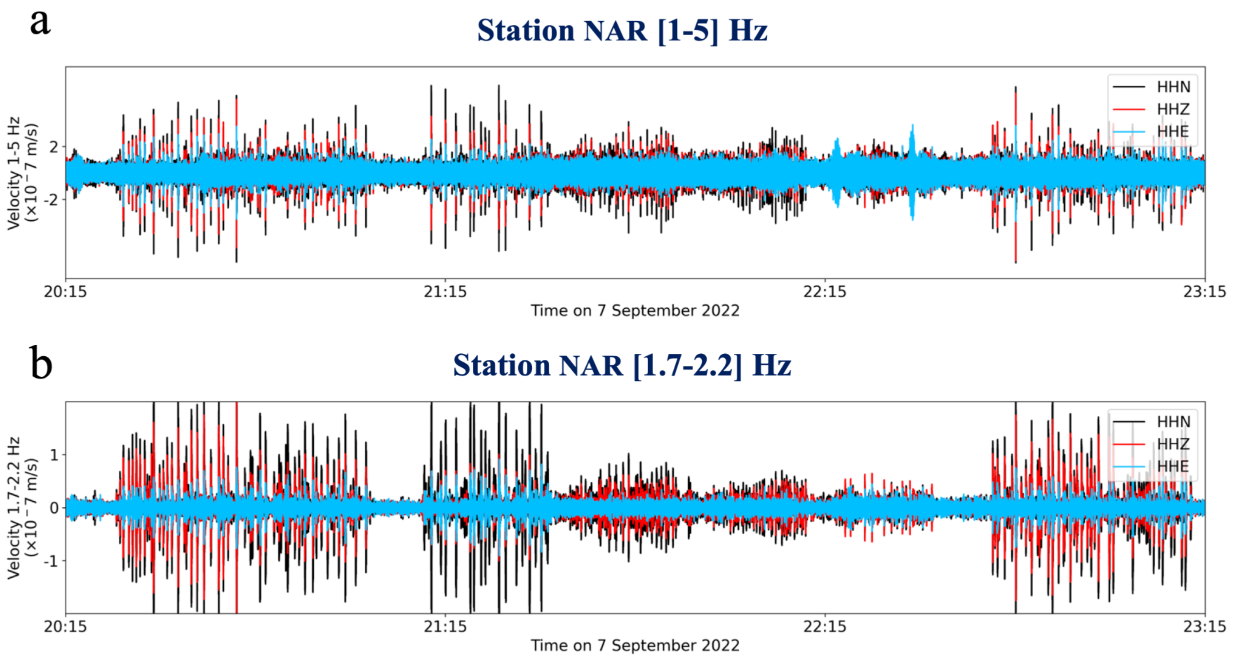

Fig. S5. Seismic waveform showing low-frequency episodes. a & b. The seismic waveforms of episodes in the frequency bands [1-5] Hz and [1.7-2.2] Hz, respectively. Episodes exhibit higher energy on the HHN component.

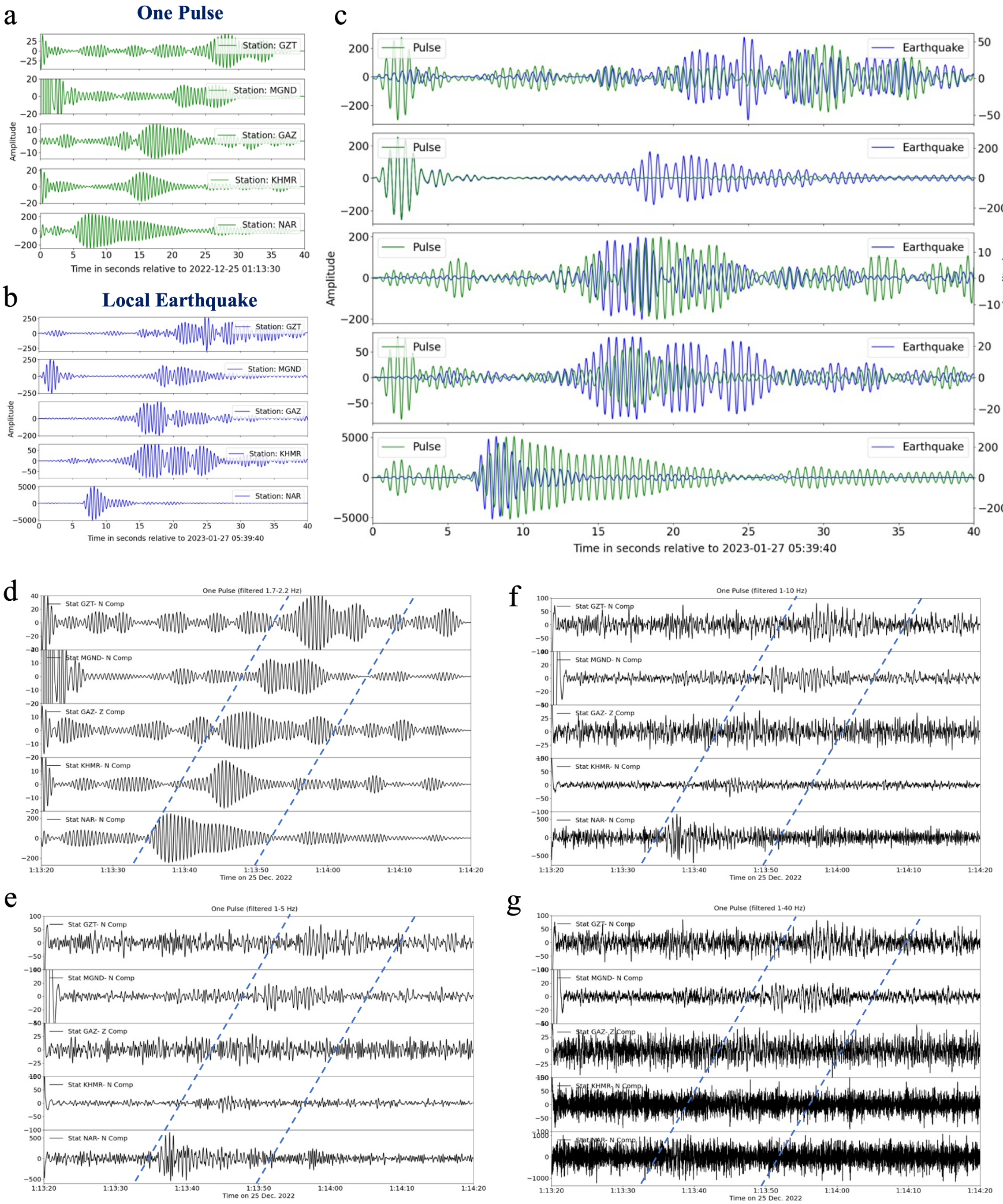

Fig. S6. Moveout comparison of a local earthquake and a pulse. a & **b.** The moveout of a pulse and a local earthquake, respectively, on the HHN component of the NAR station, filtered between 1.7 and 2.2 Hz. **c**. Comparison of the pulse and local earthquake moveouts. **d-g.** These panels show the pulse across 5 stations in different frequency bands, illustrating the actual frequency content of the pulse across these bands.

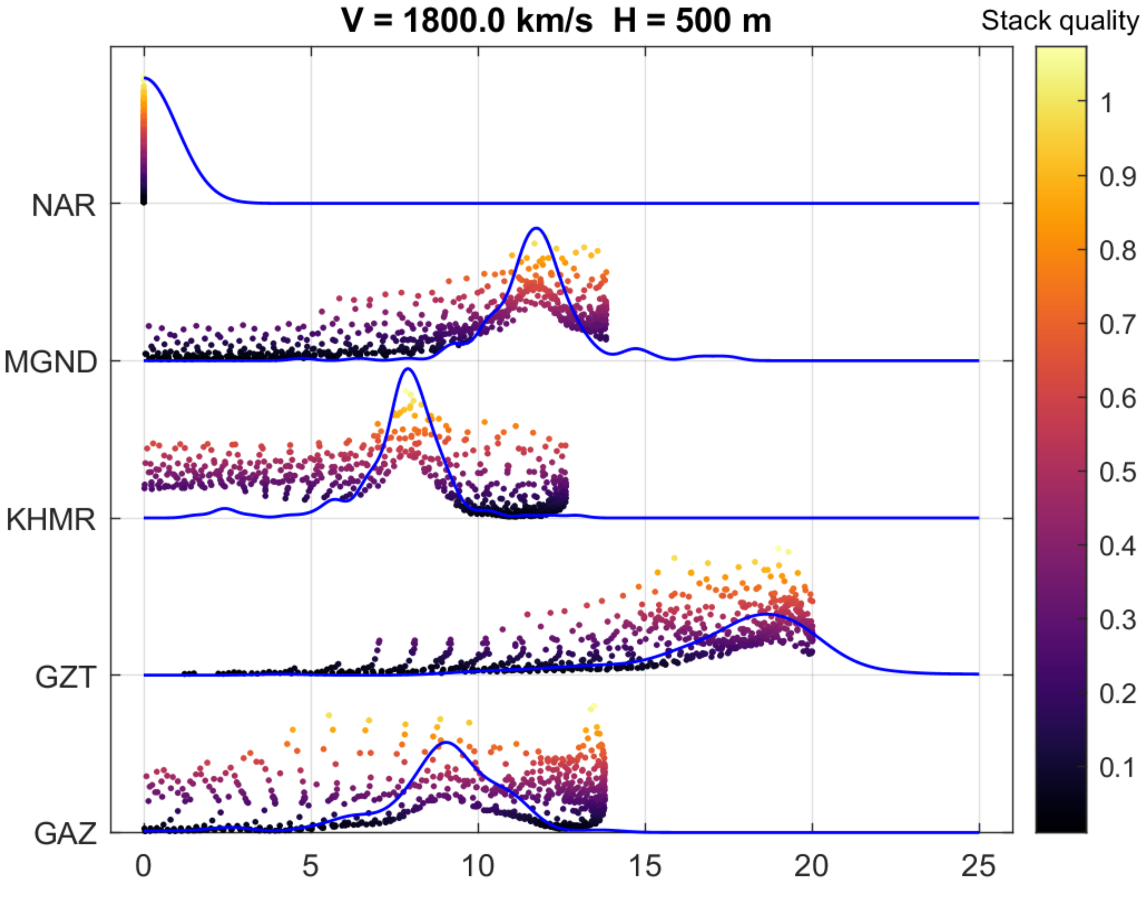

Fig. S7. Comparison of the measured and modeled arrival times. Measured arrival times at different stations are normalized to station NAR and presented as probability distribution functions (PDFs) (solid black lines). The modeled arrival times for a grid of test epicenters at an altitude of +500 meters are forward modeled assuming a constant propagation velocity of 1800 m/s, normalized to station NAR, and presented as dots. The dot color reflects the fit between observed and modeled picks (warmer color = better fit).

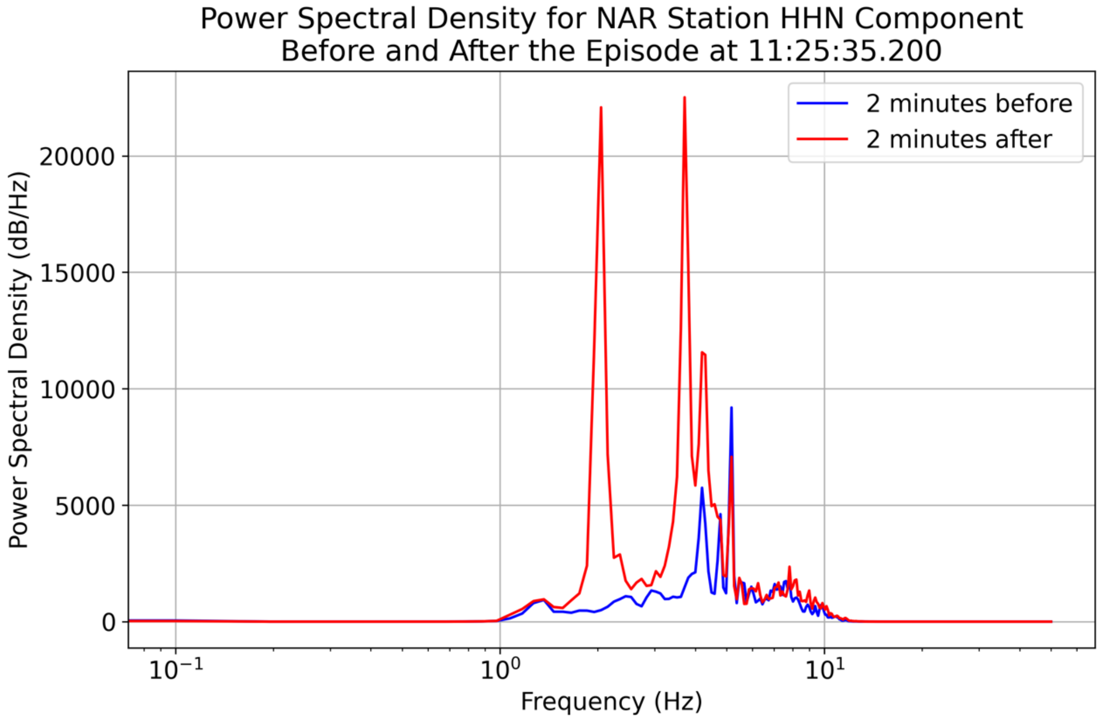

Fig. S8. Power Spectral Density (PSD) comparison of the seismic signal before and during the episode. The blue curve represents the PSD for the 2-minute window before the episode, while the red curve corresponds to the 2-minute window during the episode. Both traces were bandpass filtered between 1 and 10 Hz.

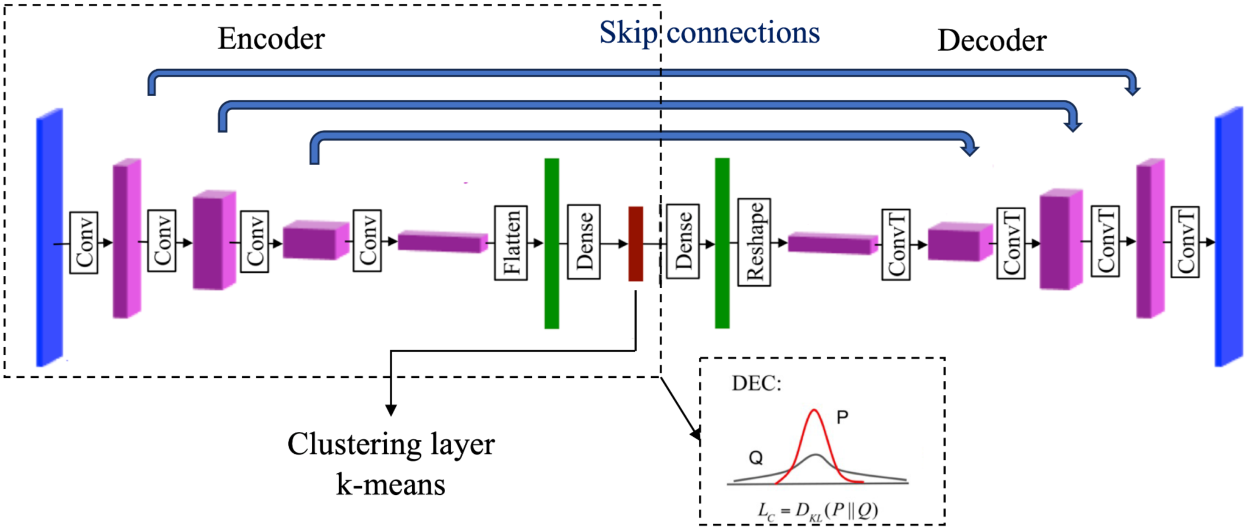

Fig. S9. Network architecture. The encoder and decoder are composed of convolutional fully connected layers and skip connections. The clustering loss (L_c) is shown in the figure.

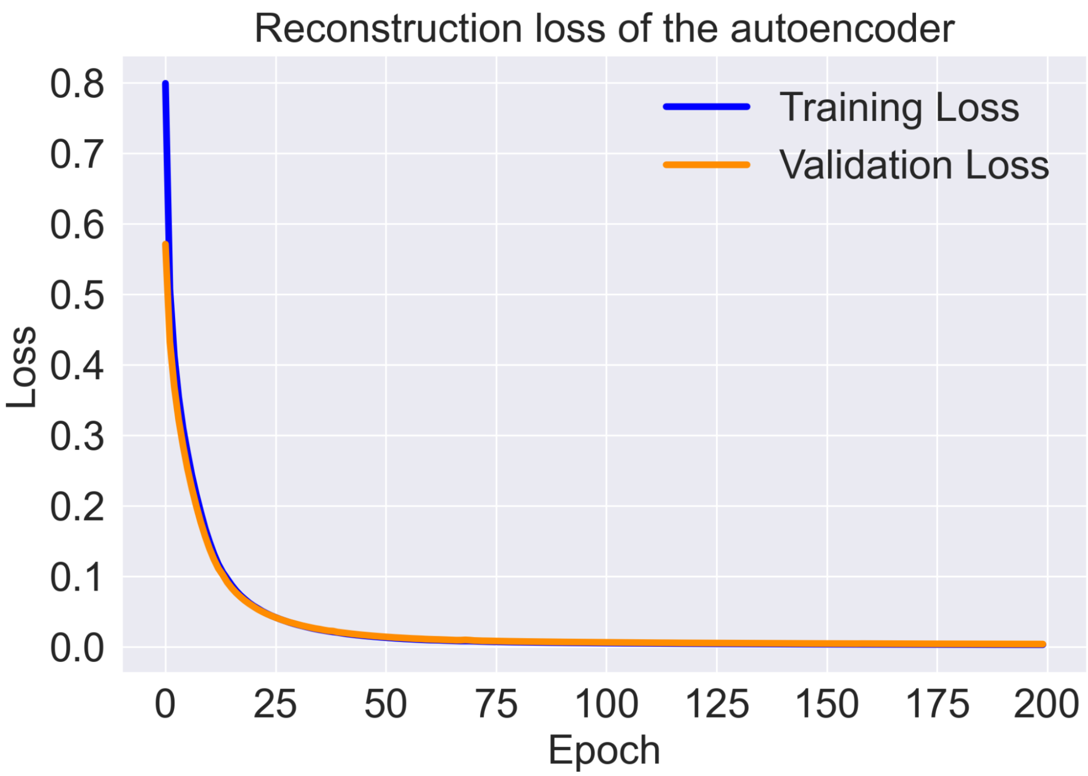

Fig. S10. Training and validation losses. Both training and validation losses exhibit an exponential decrease throughout autoencoder training.

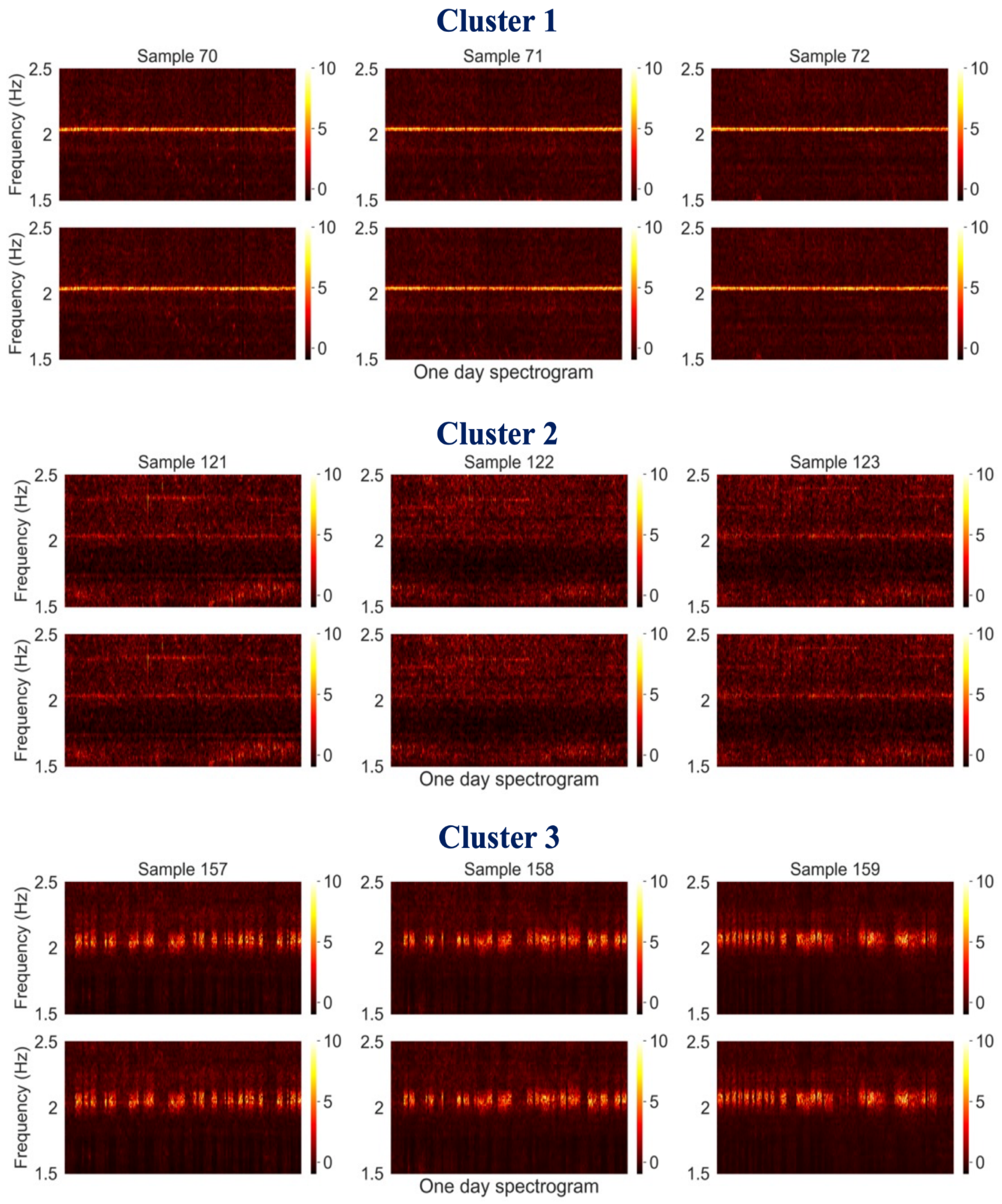

Fig. S11. Example of autoencoder's inputs and outputs. The autoencoder's proficiency in reconstructing the input from the latent spectrogram is demonstrated in this figure. In each cluster, the first row shows the input sample and the second row shows the reconstructed spectrogram in the decoder.

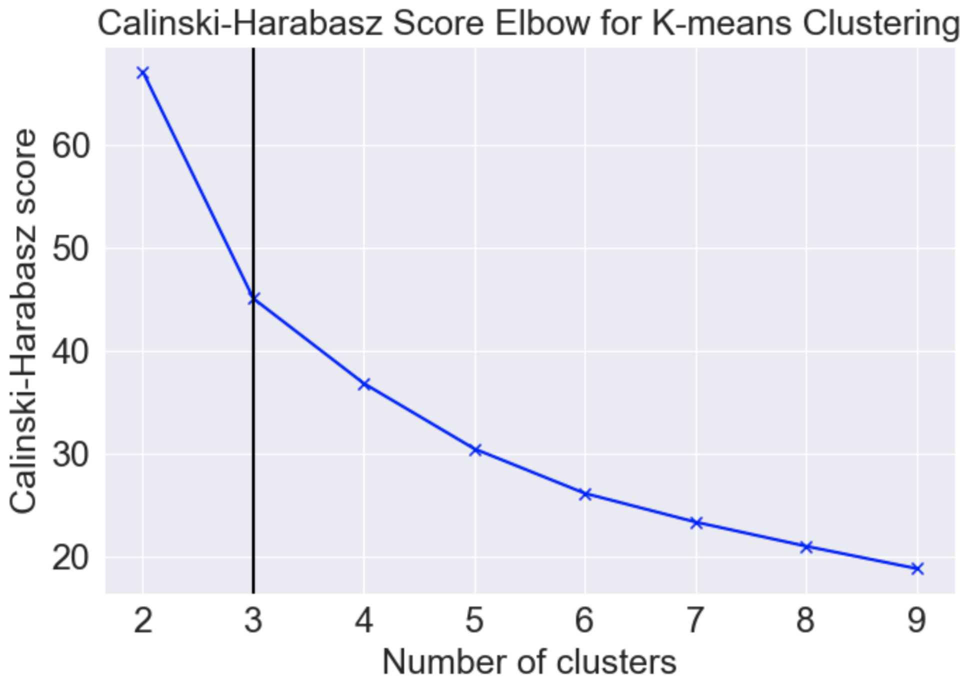

Fig. S12. Choosing the optimal number of clusters. The number of clusters k=3 is chosen by calculating the Calinski-Harabasz score.
